# Impact of COVID Pandemic and Hybrid teaching on Final year MBBS students’ End of clerkship Exam performance

**DOI:** 10.12669/pjms.38.1.4645

**Published:** 2022

**Authors:** Sahira Aaraj, Fareeha Farooqi, Nadia Saeed, Sabeen Khan

**Affiliations:** 1 Dr. Sahira Aaraj Assistant Professor of Pediatrics Shifa College of Medicine, Islamabad, Pakistan; 2 Dr. Fareeha Farooqi Associate Professor of Surgery Shifa College of Medicine, Islamabad, Pakistan; 3 Dr. Nadia Saeed Associate Professor of Medicine Shifa College of Medicine, Islamabad, Pakistan; 4 Dr. Sabeen Khan Assistant Professor of Pediatrics Shifa College of Medicine, Islamabad, Pakistan

**Keywords:** Assessment, OSCE, MBBS Clerkship, Hybrid Learning

## Abstract

**Background & Objectives:**

The novel coronavirus (SAR-CoV-2) pandemic has revolutionized medical education worldwide. Most medical schools have adopted the online teaching and assessments. Students attending modified clerkships and assessments under the stress of the pandemic, perform and score differently when compared to normal clerkships. We aimed to identify the impact of COVID-19 on final year MBBS students’ EOC (End of Clerkship) examination by comparing them with their scores prior to the COVID and with scores of the previous final year.

**Methods:**

This cross-sectional study was conducted at Shifa College of Medicine. Final year MBBS students’ scores of years 2019 and 2020 were included. Students’ EOC MCQ and OSCE scores were compared in pre-COVID and COVID affected rotations of the same year and with the previous year (2019). Data were analyzed in SPSS version 21, means scores were calculated, and one-way ANOVA was applied. Pearson correlation was calculated for correlation assessment of MCQ and OSCE scores.

**Results:**

There were 118 students. The mean EOC, OSCE, and MCQ scores in rotations one to four were 72.8±6.4, 73.3± 8.1, 71.6± 7.4, 72.7± 6.7 and 44.4± 8, 47.2± 8.4, 46.1± 8.2, 48.8± 8.1, respectively. One-way ANOVA results before and after COVID lockdown were statistically insignificant (p=0.3) for OSCE and significant for MCQ in the final year class of 2020 (p=0.001). The Pearson correlation assessment between MCQ and OSCE scores (n=416) had a significant positive correlation (r=0.42, p=0.000). The overall comparison between scores of the final year class of 2019 and 2020 showed significant improvement in Surgery and Obstetrics/Gynae scores in 2020.

**Conclusion:**

During the COVID pandemic, the final year students’ performance in EOC MCQ and OSCE over all remained unaffected.

## INTRODUCTION

The COVID-19 pandemic has impacted all aspects of our lives, including medical education.^1^ In order to follow the lockdown and stay-at-home policies, schools, colleges, and universities were shut down across the world.^2^ Subsequently, the educational environment took a paradigm shift in many medical schools, both nationally and internationally. Online and hybrid learning and assessment became a key component to deliver a medical curriculum while maintaining social distance. For the last many years, online and hybrid teaching has been used as a part of face-to-face teaching by many universities in developed countries.3,4 Assessment is an essential component of teaching and learning, as it establishes the achievement of the learning outcomes of a course. It can be divided into “assessment of learning” (summative) and “assessment for learning” (formative).1,4 Summative assessment is used for pass/fail decisions, whereas formative assessment is valuable for providing feedback. Computer-based assessment is in place for the last many years; however, online assessments have been less practiced.5 This is because of the issues of validity, reliability, and fairness.5,6 In medical schools it is crucial to assess the skills along with knowledge. Skill can be tested through OSPE, OSCE, mini-CEX, viva, and short and long cases.4,5 During pre- COVID time, these domains of learning were assessed face to face.5 However, due to the current pandemic, the majority of the assessments were either canceled, postponed, or adjusted in other formats.^7^,^8^ Because of the uncertainty of the longevity of the pandemic many of the medical schools took online modified clinical examinations for the low stake exams.

The final year of MBBS is a high stake and defining year in the life and career of a medical student.9 The tumultuous year had a significant impact on medical education and more so for the graduating class of MBBS.10 Lockdown was imposed in March 2020 to limit the spread of infection, closing down universities and medical colleges. This resulted in the suspension of on-campus and in-hospital learning activities. However, medical education swiftly moved to online platforms like Zoom, Microsoft Teams, Skype and, Google meet.11 This enabled lectures and small group teaching to continue, while the formative assessments assisted for a continued improvement.

Our university (Shifa Tameer e Millat University), also conducted EOC written final year assessments online, partly. The Rest of the EOC assessments were taken on-campus when it was opened as per government directive. Furthermore, the on-campus clerkships were squeezed to half the total of pre-COVID duration, thus reducing time for the clinical exposure and hands-on practice. Keeping this background in mind we aimed to identify the impact of the COVID-19 on final year medical students’ EOC scores by comparing these scores with the pre-COVID scores of these students, and with the scores of previous final year clerkships. This study will provide a way forward for planning and improvement of our hybrid model and assessment strategies during this unpredictable time.

## METHODS

This cross-sectional study was carried out in SCM after approval from IRB (IRB 371-1191-2020) and the duration of the study was six months. Final year MBBS students at SCM undergo four clerkship rotations of nine weeks’ duration each. These four groups rotate in disciplines of Surgery and allied, Medicine and allied, Obstetrics/Gynae, and Pediatrics. Each group comprises 25 to 30 students. In each rotation, students have real patient encounters along with interactive sessions in the form of morning reports and topic presentations. Each group rotates in operation theaters, inpatient and outpatient departments. In the ninth week, assessment is taken including written exam and OSCE. On 15th March 2020, our medical college was closed for on-campus activities on government directives for pandemic control. At that time our first rotation of clerkships had been completed along with its assessments and the second rotation of clerkships was ongoing and had completed eight weeks of rotations. The college closure was followed by online academic activities. These online activities encompassed majorly the theoretical components of the curriculum and limited possible online clinical exposure. When the situation improved, on-campus clerkship activities were resumed on the 15^th^ of October 2020 for a period of 1.5 months with due considerations of COVID-related standard operating procedures. The pending second rotations’ EOC assessments were taken and were followed by squeezed clinical rotations of third and fourth clerkships. These squeezed clinical rotations were of four weeks duration each, mainly focusing on patients’ encounter and clinical exposure. Each squeezed rotation was followed by EOC OSCE and written assessments.

The objective of this study was to compare the OSCE and MCQ scores of the rotations which were assessed in different circumstances of pandemic stress, compromised teaching, and reduced on-campus clerkship activities, with the pre-pandemic students’ scores. For this purpose, we compared the scores of the final year class of 2020 obtained during the pandemic with the scores obtained by the same class in pre-pandemic rotations. In addition, another comparison was made between the overall EOC MCQ and OSCE scores of the class of 2020 and 2019 for further analysis of our objective. The students who did not appear in assessments were excluded from the study. The data for the two years were collected and analyzed in SPSS version 21. The mean scores with standard deviations were calculated for each rotation and the one-way analysis of variance (ANOVA) was applied to determine whether there were any statistically significant differences between the means of these scores. The correlation between OSCE and MCQ scores in different rotations was assessed by Pearson correlation for class 2020.

## RESULTS

### OSCE and MCQs Scores Analysis of Final year batch 2020:

There were 118 students of the final year MBBS batch 2020. The number of students included for corresponding data analysis is shown in tables.

### One-Way ANOVA Results:

The number of students’ scores included in rotations 1, 2, 3, and 4 were 111, 110, 114, and 116, respectively. The mean EOC OSCE and MCQs scores with standard deviations in rotations one to four were 72.8±6.4, 73.3± 8.1, 71.6± 7.4, 72.7± 6.7 and 44.4± 8, 47.2± 8.4, 46.1± 8.2, 48.8± 8.1, respectively.

Overall, the differences between mean OSCE scores of different rotations were statistically insignificant (p=0.3). The Tukey post hoc test did not show any statistically significant difference between these rotations scores. However, there was a highly significant difference in the EOC mean MCQ scores (p=0.001). The Tukey post hoc test revealed a highly significant difference between first and fourth rotations (p= 0.00) and a significant difference between third and fourth rotations scores (p=0.05).

The impact of the pandemic was assessed individually for each discipline in the four rotations. The mean score differences in EOC OSCE and MCQs, in various disciplines showed few significant values, as shown in Table-I. However, in general, any specific trend could not be established in this analysis.

**Table I:** One-way ANOVA: Final year end of clerkship OSCE and MCQ scores in various disciplines (class of 2020).

Disciplines	Rotations	Number of students (n)	OSCE scores (Out of 100)		MCQs scores (Out of 70)	

			Mean scores with SD	p values	Mean scores with SD	p values
Surgery	1	28	71.6±6.6	0.10	38.4±8.1	0.61
	2	27	73.7±7.5		38.8±5.8	
	3	28	73.5±3.9		40.7±5.7	
	4	29	75.3±4.1		39.7±7.2	

Pediatrics	1	28	75.8±5.1	0.00	50.4±7	0.002
	2	27	64.1±5.8		55±5.7	
	3	29	63.6±7.4		53.9±7.1	
	4	29	68.8±5.7		49.3±5.2	

Obstetrics and Gynae	1	29	70.8±6.06	0.016	42.6±5.4	0.00
	2	29	75.9±5.5		44.4±6.2	
	3	28	72.0±6.3		41.4±6.3	
	4	29	71.1±6.8		52.9±6.3	

Medicine	1	26	73.2±7.1	0.001	46.3±6.4	0.00
	2	27	79.2±4.9		50.7±5.7	
	3	29	77.2±3.4		47.9±5.9	
	4	29	75.5±5.7		53.4±4.9	

### Pearson Correlation Analysis of EOC MCQs and OSCE of class 2020:

The Pearson Correlation Analysis between MCQs and OSCE scores (n=416) of this final year batch had a highly significant positive correlation (r=0.42, p=0.000).

### OSCE and MCQs Scores Analysis of Final year batch 2020 and 2019:

The comparison of students’ performance with that of the previous final year batch of 2019 demonstrated a significant improvement in the mean scores of OSCE and MCQs in Obstetrics/Gynae, Surgery, and Medicine in 2020 as shown in Table-II. The exception was Pediatrics MCQs, in which scores dropped significantly in 2020. The rest of the analysis remained insignificant.

**Table II:** Comparison of students’ performance variance in years 2019 and 2020, end of clerkship OSCE and MCQ.

Disciplines	Years	Number of students (n)	OSCE scores (Out of 100)		MCQ scores (Out of 70)	

			Mean scores with SD	p values	Mean scores with SD	p values
Surgery	2019	117	68.9±9.2	0.00	40.4±6.6	0.26
	2020	112	73.5±5.8		39.4±6.8	
Pediatrics	2019	117	68.8±10.9	0.53	56.4±8.4	0.00
	2020	113	68.1±7.7		52.2±6.7	
Obstetrics and Gynae	2019	117	68.7±8.9	0.00	42.6±6.3	0.002
	2020	115	72.5±6.8		45.4±7.5	
Medicine	2019	117	75.2±9.9	0.28	47.8±7.4	0.039
	2020	111	76.3±5.7		49.7±6.3	

## DISCUSSION

Final year MBBS student of the class 2020 spent major time on virtual learning through online classes. Reduced time was spent in the hospital for bedside teaching, practicing clinical skills, and direct patient interactions. Considering the novelty of the online teaching process both for faculty and students it was imperative to study the impact of students’ performance in EOC exams both in OSCE and MCQs, as these assessment methods are equally important to evaluate students’ knowledge and skills.^12^ EOC exam is a vital component in preparing students for the final professional MBBS exams and also contributes towards the annual scores.^13^,^14^

The mean scores improved in Medicine, Pediatrics, and Obstetrics/Gynae from the first EOC MCQ assessment to the second EOC assessment. A reason may be that the students of the second rotation had undergone normal clerkship teaching before COVID lockdown and they had additional time for preparing for the EOC exams. A similar trend is observed between scores of third and fourth rotations, both were conducted after online teaching followed by a reduced four-week rotation on campus. The fact might be that lockdown and restriction of social activities positively influenced the learning process as students had more time in hand for studies. A recent study about the COVID impact from the same institute revealed that almost 60% of students agreed that they had more time to study. 15 A similar trend has been observed in the literature reporting students’ performance in higher studies.^16^

The OSCE scores also improved in Surgery, Obstetrics/Gynae, and Medicine when compared between pre-COVID and resumed rotations (Table-I). While those of pediatrics decreased slightly. Both surgery and Obstetrics/Gynae are the disciplines where students also attend operations and surgeries along with inpatient and outpatient sessions. So, in usual circumstances, they have more chance to see the operative management of patients but they get less time for theoretical knowledge and self-study. Probably while delivering online sessions more focus was given on the theoretical component leading to scores improvement in these two disciplines. Again, this could be the reason for improved mean scores for the year 2020 in disciplines of Surgery and Obstetrics/Gynae.

The clinical skills learning and assessment are quite different from the theory. The hands-on practice and direct patients’ interaction have a distinct impact on skills development rather than discussing it online. 17,18 We found that the OSCE scores of pediatrics clerkship deteriorated in this situation where students had a compromised clerkship rotation and a lesser exposure to the patients (p=0.00), emphasizing that students needed more time for bedside learning. Except for Pediatrics, students showed improvement in their OSCE performance during the COVID pandemic.

As compared to 2019 results (Table-II), scores in clinical OSCE improved in Surgery and Obstetrics/Gynae (p< 0.005), despite of having a difficult, interrupted, and hybrid course of studies in 2020. Similar results were observed by Prighoff et al.^19^ students were given maximum clinical exposure once they were on campus after a lockdown to make up for their deficiency. The mean scores are comparable in Pediatrics and Medicine OSCE. While MCQ scores showed improvement in Obstetrics/Gynae, and Medicine and remained static in Surgery (<0.005). Despite all the mayhem and disruption to on-campus teaching, the students have shown good results taking the opportunity of online teaching positively. Our hybrid model has likely improved self-directed learning among our students. Stress and fear of the disease might have affected the concentration and performance of some of the students. Hamza et al and Lorenzo et al reported that the COVID-19 pandemic has induced stress and changes in medical students’ educational attitudes and strategies. They found 22.3% of students perceived severe stress as they did not prefer online learning.20,21 Uncertain circumstances and multiple time extensions of lockdown also affected the students negatively. Birch and Byung et al found that exam disruptions had a statistically significant impact on preparedness for both OSCEs and written examinations (p< 0.005).6,22

### Limitation of the study:

We could not include students’ perspectives in this study about online teaching and learning. Only final year students were included while the first three years’ students could not be included due to the limited objectives of the present study.

## CONCLUSION

During the stressful time of the COVID pandemic lockdown**,** our students’ performance, in general, remained unaffected in terms of EOC MCQ and OSCE scores, indicating that our hybrid model and online teachings brought a positive impact on our students and can be followed further.

### Author`s Contribution:

**SA:** Basic idea of study. Manuscript writing and editing

**FF:** Manuscript writing. Final editing

**NS:** Statistical analysis. Interpretation of the results. Draft revision

**SK:** Interpretation of results, manuscript writing

All authors are responsible and accountable for accuracy or integrity of work and give final approval of the version.
